# Metabolic syndrome and renal cell carcinoma

**DOI:** 10.1186/1477-7819-12-236

**Published:** 2014-07-29

**Authors:** Gui-Ming Zhang, Yao Zhu, Ding-Wei Ye

**Affiliations:** 1 Department of Urology, Fudan University Shanghai Cancer Center, No. 270, Dongan Rd, Shanghai 200032, China; 2 Department of Oncology, Shanghai Medical College, Fudan University, Shanghai, China

**Keywords:** Metabolic syndrome, Renal cell carcinoma, Insulin resistance, Mechanism

## Abstract

**Background:**

Metabolic syndrome (MS) is a cluster of metabolic abnormalities, which has been regarded as a pivotal risk factor for cardiovascular diseases. Recent studies focusing on the relationship between MS and cancer have recognized the significant role of MS on carcinogenesis. Likewise, growing evidence suggests that MS has a strong association with increased renal cell carcinoma (RCC) risk. This review outlines the link between MS and RCC, and some underlying mechanisms responsible for MS-associated RCC.

**Materials and methods:**

A National Center for Biotechnology Information PubMed search (http://www.pubmed.gov) was conducted using medical subject headings ‘metabolic syndrome’, ‘obesity’, ‘hypertension’, ‘diabetes’, ‘dyslipidemia’, and ‘renal cell carcinoma’.

**Results:**

This revealed that a variety of molecular mechanisms secondary to MS are involved in RCC formation, progression, and metastasis. A deeper understanding of these molecular mechanisms may provide some strategies for the prevention and treatment of RCC.

**Conclusions:**

In summary, there is a large body of evidence regarding the link between MS and RCC, within which each component of MS is considered to have a close causal association with RCC.

## Review

### Background

Metabolic syndrome (MS) has become an almost ubiquitous severe health issue across the globe. It is estimated that more than 40% U.S. residents over the age of 60 years have MS [1], with a prevalence of approximately 25% in European and Latin populations [2,3]. Over recent decades there has been an alteration of dietary pattern and lifestyle in Chinese people, and subsequently an elevated morbidity associated with MS year by year. According to an analysis of data in 2005, up to 20% Chinese adults suffered from MS [4]. The concern regarding MS was primarily focused on its contribution to increased cardiovascular disease and type 2 diabetes mellitus risk. Recently, the relationship between MS and cancer has been highlighted by the observation that MS is associated with high morbidity and mortality for certain types of cancer. This review summarizes evidence in support of the relationship between MS and renal cell carcinoma (RCC) and possible underlying mechanisms and therapeutic interventions.

### Definition of metabolic syndrome

MS was first described using the term ‘syndrome X’ by Reaven in 1988 [5], with insulin resistance (IR) as the common and elementary denominator. It comprises a cluster of metabolic abnormalities, each of which is an important atherosclerotic risk factor. With a deeper understanding of MS, more sophisticated mechanisms have been revealed, and IR is recognized as one of the most significant factors. Because IR is not easily measured clinically, it is not included in all of the definitions developed by different organizations, whereas abdominal obesity, dyslipidemia, hypertension, and impaired glucose regulation have been adopted in almost all of the diagnostic criteria. For example, NCEP/ATP III (National Cholesterol Education Program/Adult Treatment Panel III) sets three of five of the following criteria as necessary for a diagnosis of MS: fasting glucose ≥110 mg/dl; hypertriglyceridemia ≥150 mg/dl; low high-density-lipoprotein (HDL) levels - male <40 mg/dl, female <50 mg/dl; abdominal obesity - male >102 cm, female >88 cm waist circumference; hypertension, ≥130/≥85 mmHg. In 1998, the World Health Organization (WHO) recommended a unifying definition and revised this in the following year: impaired glucose regulation and/or hyperinsulinemia (fasting glucose ≥110 mg/dl; post-prandial glucose >140 mg/dl; fasting serum insulin: third quartile for control group) and two or more criteria are necessary: (1) hypertension, ≥140/≥90 mmHg; (2) hypertriglyceridemia, ≥150 mg/dl; low HDL levels - male <35 mg/dl, female <39 mg/dl; (3) abdominal obesity - waist:hip ratio, male >0.90, female >0.85, and/or body mass index (BMI) ≥30 kg/m^2^; (4) microalbuminuria, ≥20 μg/min.

### MS and cancer

There is increasing evidence to indicate that MS might play an important role in the etiology and progression of certain types of cancers. The association of MS with liver, pancreatic, gastric, colorectal, bladder, prostate, endometrial, cervical, and postmenopausal breast cancers have all been reported [6-14]. A systematic review published in 2012 showed that MS was associated with pancreatic and rectal cancer, and these associations were stronger in women than in men. Additionally, associations were different for ethnic groups: stronger associations were observed in Asian populations for liver cancer, in European female populations for colorectal cancer and in U.S. Caucasian populations for prostate cancer [15]. Cancer patients with concomitant MS seem to have a worse prognosis. Buschemeyer et al. reported that obese patients with prostate cancer were more likely to suffer from high-grade and aggressive cancer [16]. Compared with those with high cholesterol levels, men with normal or borderline levels were less likely to develop high-grade prostate cancer, particularly when considering cases in which the cancer was confined to the pancreas [17]. A conclusion that MS is a risk factor for lethal prostate cancer has also been reported [11]. Further, the rates of liver metastasis and tumor recurrence were higher in patients with colorectal cancer accompanied by MS [18], and Pasanisi et al. observed a higher recurrence rate in breast cancer patients with concomitant MS [19].

### MS and renal cell carcinoma

Renal cell carcinoma (RCC), a common type of urologic tumor, accounts for 2% to 3% of all malignancies. According to data provided by the WHO, the morbidities of RCC appear to be showing an upward trend in recent years in a range of regions and ethnicities, except for in Scandinavia [20]. Although the etiology of RCC remains unclear, previous research has identified smoking and diet as risk factors. Moreover, data from a large set of studies suggest a significant association between RCC and the major components of MS, among which obesity and hypertension have been listed as confirmed etiological factors in the guidelines by many organizations, for example, the European Association of Urology, the American Urological Association, and the Chinese Urological Association.

### Obesity and RCC

Accumulating evidence indicates that, as an independent risk factor, obesity is significantly associated with RCC [21]. A study whose conclusion was published in the *British Journal of Cancer* analyzed 22 independent clinical studies available in MEDLINE from 1966 to 1998. By means of quantitative summary analysis, the authors reported that the link between MS and RCC was stronger among men with higher BMI, and the relative risk for men and women together was 1.07 (95% CI: 1.05-1.09) per unit of increase in BMI (one unit of increase in BMI corresponds to 3.1 kg for a man of an average height of 1.77 m, and to 2.7 kg for a woman of an average height of 1.64 m) [22]. Leiba and his colleagues conducted a study comprising 19,576,635 person-years of follow-up and drew a conclusion that being overweight in late adolescence was a substantial risk factor for RCC, and European origin was independently associated with excess risk compared with Asian or African origin [23]. Nevertheless, it is of interest that obesity seems to be a favorable factor in terms of prognosis of RCC, despite its contribution to increased RCC risk. For patients with organ-confined but not advanced RCC, being overweight improved their cancer specific survival [24]. Another study also reported that cancer-specific survival time, but not overall survival time, was notably prolonged for patients with increasing BMI (>30 kg/m^2^) who had received radical nephrectomy [25].

Some recently published studies showed that compared with subcutaneous adipose tissue, visceral adipose tissue seemed more harmful to health, and to be a better indicator than BMI of obesity. In addition to the traditional measure of waist:hip ratio, visceral fat area (VFA) measured using imaging methods has become a new research ‘hotspot’. Zhu et al. investigated the relationship between tumor grade and VFA in patients with RCC of stage T1a and found that the percentage of visceral adipose tissue was significantly associated with higher Fuhrman grade and might be an independent predictor of high grade RCC [26]. However, visceral adipose tissue is likely to play a protective role in advanced RCC patients who are receiving first-line targeted therapy, such as sorafenib and sunitinib. The fact that those patients with higher levels of VFA had a longer progression-free survival time and OS has been reported in a retrospective study [27]. The reason why obesity is associated with increased RCC risk but improved prognosis is not yet well understood. It has been speculated that the development of cachexia might be suppressed by obesity. Further studies are needed to discover the underlying biological mechanism.

### Hypertension and RCC

The conclusion that hypertension can increase the risk of RCC has been confirmed by a large set of clinical studies. A study group evaluated the relationship between hypertension and RCC by examining the health records of 363,992 Swedish men, with subsequent follow-up studies. After the first 5 years of follow-up had been excluded to reduce possible effects of preclinical disease, the researchers still found a direct association between higher blood pressure and a higher risk of RCC [21]. Colt et al. also performed a clinical study in the U.S. and found hypertension doubled RCC risk, with OR of 1.9 (CI: 1.5-2.4) for Caucasians, and 2.8 (CI: 2.1-3.8) for African-Americans [28]. A similar conclusion was also reported in a Chinese population-based study [29]. Subtle changes of renal function prior to clinical hypertension may make the kidney more susceptible to carcinogenesis, and some angiogenic and other growth factors that are involved in hypertension may also participate in renal carcinogenesis and progression.

It is noteworthy that some interventions aiming to reduce hypertension may also increase the risk of RCC, of which diuretics merit further investigation. As the first line antihypertensive drugs that can reduce morbidity and mortality of cardiovascular disorders, diuretics are widely administrated owing to their satisfactory effect and relatively low expense. However, emerging evidence suggests that the long-term use of diuretics may be associated with RCC [30], being especially significant for women [31]. Nevertheless, because this conclusion is still controversial and the reason for it remains unclear, large-scale studies to corroborate the conclusion and clarify the mechanisms are warranted.

### Diabetes and RCC

The relationship between diabetes and cancer is quite close, because it is presently considered that there may exist ‘common soil’ responsible for both diabetes and cancer. In other words, some risk factors for diabetes likewise render people more susceptible to cancer. There are also numerous epidemiological studies focusing on the link between diabetes and RCC, showing an elevated cancer morbidity and mortality in diabetes patients. Lindblad and colleagues conducted a large scale follow-up study (1 to 25 years) in a total of 153,852 patients with a diagnosis of diabetes in Northern Europe. After exclusion of the first year of observation, they found that standardized incidence ratios (SIRs) were 1.7 (95% CI: 1.4-2.0) in women and 1.3 (95% CI: 1.1-1.6) in men, and standardized mortality ratios (SMRs) were 1.9 (95% CI: 1.7-2.2) in women and 1.7 (95% CI: 1.4-1.9) in men. Thus, both morbidity and mortality of RCC increased in patients with diabetes in comparison with the general population [32]. Another study with a follow-up of 32 years performed in the U.S. reported type 2 diabetes was independently associated with an increased risk of RCC in women [33].

Another current research ‘hotspot’ concerning the impact of anti-diabetic drugs on tumor development provides indirect evidence about the role of diabetes on the etiology of cancers. Insulin can promote mitosis and cell proliferation, which results in the concern about its carcinogenic effect. However, no compelling evidence that exogenous insulin or insulin analogues may elevate cancer risks has so far been presented. Many studies have revealed the inhibitory effect of metformin on many kinds of tumor cells, including RCC cells. Metformin, one of the commonly used oral anti-diabetic drugs, is able to inhibit the growth of RCC cells *in vivo* and *in vitro*, through induction of apoptosis and G0/G1 cell cycle arrest [34,35]. No consensus as to the effect of sulfonylureas on cancer has been reached so far. Deeper and more systematic work needs to be done to clarify the relationship between anti-diabetic drugs and tumor.

### Dyslipidemia and RCC

Lipid profiles include cholesterol (CH), triglyceride (TG), and some lipoid constituents, such as phospholipids, of which CH and TG are clinically important. Only in the form of lipoprotein, for example, low-density-lipoprotein (LDL) and HDL, can CH and TG be transported to the tissues and be metabolically active. There are many studies validating the contribution of dyslipidemia to carcinogenesis of various cancers. An elevated risk of esophageal cancer and colon cancer was observed in people with high levels of TG [36,37], and hypercholesterolemia has been suggested as a risk factor for rectal cancer [36]. High serum CH and TG levels raised the risk of prostate cancer and low HDL levels were associated with breast cancer, lung cancer, and non-Hodgkin’s lymphoma [38-41].

Clear cell RCC, the most common type of renal malignancy, is characterized histologically by sterol storage in tumor cells, which prompts some abnormality in lipid metabolism which plays an important role in the formation and progression of RCC. However, little research has been conducted which directly focuses on the association between RCC and lipid disorders. In a prospective cohort study, Van Hemelrijck et al. reported that TG was the only lipid component for which a statistically significant association was observed with RCC [42], whereas another prospective study reported a decreased risk of RCC associated with higher serum CH levels, albeit with marginal statistical significance [43]. Statins (inhibitors of 3-hydroxy-3-methylglutaryl coenzyme A reductase) that are used for the treatment of lipid disorders, especially hypercholesterolemia, appear to protect against the development of RCC. Horiguchi et al. reported that fluvastatin had a notable inhibitory effect *in vitro* on tumor growth, invasion, angiogenesis, and metastasis of RCC cells [44], which provided indirect evidence of the relationship between dyslipidemia and RCC. However, given the relative paucity of laboratory and clinical research compared with that relating to obesity, hypertension, and diabetes, further studies regarding the effect of dyslipidemia on RCC development are warranted.

### Mechanisms

The mechanisms that underlie the role of MS on RCC carcinogenesis are complicated, involving insulin resistance (IR), inflammation, angiogenesis, cell-stroma interaction, and many other important aspects. A sophisticated network including various factors and signaling pathways is composed of these interrelated components, regulating the cross-talk between MS and RCC.

### Insulin resistance

Hyperinsulinemia and IR are considered the primary basis of the metabolic impairment associated with MS. Insulin is one of the most important anabolic hormones that is able to stimulate cell proliferation. Harmful local and systemic reactions, including intracellular lipid accumulation in adipocytes, mitochondrial and endoplasmic reticulum stress, and IR, can be induced by obesity, accompanied by changes of circulating factors such as adipokines, free fatty acids and a variety of inflammatory mediators.

The insulin-like growth factor (IGF) family and the changes of its components resulting from IR play a crucial role in tumor formation and progression. The IGF family consists of IGF-1, IGF-2 and their receptors, IGF-1R and IGF-2R, as well as six types of IGF binding proteins (IGFBPs), IGFBP-1 to 6. IGF-1 and IGFBP-3 are important members of the IGF family, and have been investigated in many studies to understand their role in RCC carcinogenesis. Rasmuson et al. evaluated the prognostic information of serum IGF-1 in 256 patients with RCC, and reported that IGF-1 did not correlate with tumor stage or grade, whereas tumor stage and IGF-1 levels were independent prognostic factors using a multivariable analysis. They concluded that high serum IGF-1 levels at diagnosis were related to favorable prognosis in RCC [45]. In the human RCC cell lines Caki-2 (from a primary tumor) and SK-RC-52 (from a metastasis), IGF-1 may enhance transforming growth factor-β (TGF-β) signaling and raise IGFBP-3 levels with TGF-β acting in synergy [46]. Through activation of mitogen-activated protein kinase (MAPK) and phosphatidylinositol-3 kinase (PI3K) signaling pathways, IGF-1 that is bound to IGF-1R may play a role in promotion of mitosis and cell migration, and inhibition of apoptosis [47]. Additionally, IGF-1 can stimulate tumor angiogenesis by increasing vascular endothelial growth factor (VEGF) levels [48]. IGFBP-3 may inhibit the activity of IGF-1 through competitive binding to IGF-1, because IGF-1 has a higher affinity for IGFBP-3 than for its receptors. Moreover, IGFBP-3 regulates the biological functions of IGFs, by means of controlling their transport from circulation into tissues, modulating their metabolism and clearance, and establishing their specific location on tissues and cells [49]. Although no correlation was observed between serum IGFBP-3 levels and RCC [45,50], high expression of IGFBP-3 was found in clear cell RCC tissue, and high grade (Fuhrman grades 3 and 4) clear cell RCC showed higher IGFBP-3 staining intensity than low grade clear cell RCC [51]. Considering the close link between IGFs and RCC, potential therapeutics targeting IGFs might be promising for the treatment of RCC.

### Inflammation

As a typical alteration in obesity, both hyperplasia and hypertrophy of adipocytes may lead to tissue hypoxia, and subsequently the induction of a series of pro-inflammatory cytokines. These cytokines, such as tumor necrosis factor-α (TNF-α), interleukin-6 (IL-6), IL-8, IL-10, and macrophage inflammatory protein 1 (MIP-1), have been shown to stimulate angiogenesis and promote IR, as well as being pivotal to tumor development.

Under the conditions of MS, elevated levels of reactive oxygen species (ROS) (together with pro-inflammatory cytokines and certain mediators involved in inflammation status, such as NF-κB and cyclooxygenase-2 (COX-2)) are known to affect cell apoptosis, proliferation, and invasion. It has been shown that both IL-6 and IL-10 are strongly expressed in RCC cells and stroma. Further, IL-10 levels were higher in more advanced (pT3) tumors, suggesting that IL-6 and IL-10 may be useful markers associated with the development and progression of RCC [52]. TNF-α, another vital cytokine, was found to be positively correlated with IR and waist circumference [53]. Likewise, TNF-α is capable of promoting proliferation and metastasis of RCC cells, and mediating epithelial-mesenchymal transition of RCC via GSK3β [54]. ROS directly affect RCC and induce cell apoptosis via activation of the NF-κB pathway and downregulation of COX-2 [55].

Considerable interest has been devoted to COX-2, a key enzyme in prostaglandins synthesis, and its contribution to cancer. It has been documented that COX-2 is overexpressed in various cancers, including RCC, in which it was correlated with VEGF expression [56,57]. Additionally, IGF-IR and COX-2 may produce a synergistic effect in the oncogenesis and progression of RCC [58]. Nevertheless, Kankuri et al. reported that COX-2 expression was associated with slow development of metastases, and favorable prognosis in metastatic RCC [59]. As the current first-line drug targeting VEGF pathways, sunitinib has been applied in the clinic for the treatment of advanced RCC. It has been reported that Cox-2 inhibition enhanced the activity of sunitinib in human RCC xenografts [60].

### Adipokines

Adiponectin, which is primarily secreted by white adipose tissue, functions as a regulator of glucose and lipid metabolism and energy homeostasis. In addition to overall obesity, central fat distribution is thought to be an independent negative predictor of serum adiponectin [61], and weight loss may increase serum adiponectin levels [62]. Adipose tissue hypoxia may also result in the reduction of adiponectin and further research has shown that hypoxia and TNF-α downregulate the activity of the adiponectin gene promoter [63]. The anti-tumor role of adiponectin has been ascribed in part to its anti-inflammatory and anti-proliferative effect, as well as antagonism to IR. It was found that serum adiponectin levels were adversely associated with RCC [64]. Moreover, Pinthus et al. reported that low blood adiponectin levels had a strong correlation with tumor size and metastasis of RCC, and could potentially be used as a biomarker [65]. *In vitro* experiments have demonstrated that adiponectin can inhibit tumor growth via activating AMP-activated protein kinase (AMPK), thereby downregulating mammalian target of rapamycin (mTOR) pathways [66]. Additionally, as an endogenous inhibitor, adiponectin plays an inhibitory role on tumor angiogenesis [67].

Another important adipocyte-specific protein produced predominantly by white adipose tissue is leptin, which acts centrally to mediate satiety and bodyweight [68]. Uncontrolled energy intake and being overweight take place as a result of abnormal levels and/or function of leptin. Elevated levels of serum leptin and overexpression of leptin receptors are associated with RCC invasion and progression, and activation of the extracellular signal-regulated kinases (ERK1/2) and janus kinase/signal transducer and activator of transcription 3 (JAK/STAT3) signaling pathways participates in leptin-mediated proliferation of RCC Caki-2 cells [69-71]. By suppressing apoptosis, promoting cell proliferation and upregulating VEGF via hypoxia-inducible factor-1α (HIF-1α) and NF-κB, leptin boosts carcinogenesis [72]; HIF-1α and VEGF are thus involved as crucial factors promoting RCC formation and development.

### Peroxisome proliferator- activated receptors (PPARs)

PPARs belong to the nuclear hormone receptor superfamily and consist of three subtypes: PPARα, PPARβ, and PPARγ. These ligand-activated transcription factors contribute significantly to MS and other diseases. For example, PPARα can activate fatty acid catabolism and PPARβ participates in fatty acid oxidation. With respect to PPARγ, it has been shown to improve IR, regulate adipocyte differentiation, and play an important role in inflammation, autoimmune diseases, and cancers. Many experiments delineated a high expression of PPARγ in RCC tissue. PPARγ ligands were found to inhibit human RCC cell proliferation by induction of apoptosis and G0/G1cell cycle arrest [73,74]. Pioglitazone is a highly selective type of PPARγ ligand that serves as an insulin sensitizer and is used to treat patients with type 2 diabetes. The multiple antitumor effects of pioglitazone on RCC *in vitro*, such as suppression of cell growth, induction of apoptosis and inhibition of VEGF and basic fibroblast growth factor secretion, were reported by Yuan et al. [75]. A recent piece of research indicated that 15-deoxy-Delta12,14-prostaglandin J2 (a novel kind of PPARγ ligand) exerts cytotoxic effects on RCC cells via activation of c-Jun N terminal kinase (JNK)/MAPK and Akt pathways, in addition to induction of apoptosis [76]. Furthermore, therapies targeting PPARα are applied for dyslipidemia, and PPARβ agonists may be useful to treat obesity, diabetes, and cardiovascular diseases. All of the above research demonstrates that PPARs are potent therapeutic targets for MS and cancer.

### Hypoxia-inducible factors (HIFs)

More and more evidence has reported the contribution made by HIFs to MS. Hyperplasia and hypertrophy of adipocytes may lead to local hypoxia, which subsequently upregulates HIF expression and augments macrophage infiltration [77]. In cultured adipocytes and preadipocytes, overexpression of HIF-1α was also detected when the cells were exposed to hypoxia [78]. Hyperglycemia interferes with HIF-1α stabilization, although the mechanisms are not well-defined. Conversely, unbalanced HIF-1α signaling impairs β-cells, causing insufficient insulin secretion and IR [79-82]. A study investigating the effect of HIF-1α on obesity and diabetes found the HIF-1α knockout mice manifested resistance to weight gain, while ameliorating IR [83]. Venous hypertension stimulates HIF-1 overexpression, and consequent upregulation of VEGF [84]. In an oxygen-deficient environment, activation of the HIF signaling pathway results in lipid accumulation, and downregulates PPARα expression in a HIF-1-dependent manner [85]. It has been documented that exposure to hypoxia increases IL-6, leptin and monocyte migration inhibitory factor expression of adipocytes *in vitro*, however it was also shown to decrease adiponectin expression [78].

The role of hypoxia in RCC carcinogenesis has been verified. As a response to oxygen deficiency, HIFs not only participate in energy homeostasis, such as glucose and lipid metabolism, but also have effects on tumor development, by means of regulating the cell cycle, apoptosis, and angiogenesis. HIF-1α was found to increase intra-tumor microvessel density in xenografts, as well as being overexpressed in RCC [86]. Additionally, high expression of HIF-1α seems to indicate a poor prognosis [87]. In a retrospective study examining the prognostic prediction of HIF-1α in metastatic RCC patients, researchers found that patients with high HIF-1α expression had significantly worse survival than those with low expression (median survival: 13.5 *versus* 24.4 months, respectively) [88].

High expression of HIFs in RCC is closely related to inactivation of the von-Hippel-Lindau (VHL) gene. Somatic mutations in this gene are detected in approximately 50% of sporadic RCC, while VHL hypermethylation is seen in 10% to 20% of sporadic RCC [89]. As the VHL gene product, pVHL is absent owing to the inactivation of VHL, which mimics hypoxia and elicits a constitutive up-regulation of HIF-1α, via NF-κB activation [90]. As a result of HIF-1α overexpression, several factors involved in carcinogenesis and angiogenesis, such as VEGF, platelet-derived growth factor-β (PDGF-β) and TGF-β, are upregulated. Although more comprehensive research is needed, it is believed that hypoxia acts as a common key factor in both MS and RCC; the significance of further research lies in revealing the mechanisms and developing therapeutic interventions.

### Mammalian target of rapamycin (mTOR)

mTOR, a high molecular weight serine-threonine kinase which belongs to the superfamily of phosphatidylinositol 3-kinase (PI3K)-related kinases, is involved in cell growth, proliferation, autophagy, survival, metabolism, and angiogenesis. Deregulation of the PI3K signaling pathway is a feature of various tumors, and it is estimated to account for up to 30% of all human cancers [91,92]. MS is able to trigger changes in signals upstream of mTOR, and then regulate the processes of downstream pathways via mTOR, including protein synthesis, lipid metabolism, and mitochondrial metabolism. Insulin and IGF-1 may promote cell anabolism through mTOR. If glucose regulation is impaired, abnormality of some growth factors pathways, for example, IGF-1, will result in improper activation of mTOR and/or inactivation of mTOR inhibitors, causing various pathologies, including cancer. Meanwhile, an imbalance in the mTOR pathway leads to deterioration of IR [93], resulting in a vicious cycle. In the course of inflammation, TNF-α activates IκB kinase-β (IKKβ), which then inactivates mTOR inhibitors, thereby activating the mTOR pathway [94]. Further, obesity can raise mTOR levels, which is also involved in HIF-1α translation [95]. Conversely, hypoxia activates mTOR inhibitors via AMPK, leading to blockage of the mTOR pathway [96].

Mutation of the tumor suppressor PTEN and activation of PI3K, both of which are common in many kinds of cancers, mediate hyperactivation of mTOR. In some studies, researchers have detected low PTEN expression, and high expression of PI3K/mTOR, in clear cell RCC [97,98]. Further, high PI3K/mTOR expression seems to be associated with poorer prognosis [99,100]. Given the importance of mTOR for RCC, great progress has been made in targeting mTOR. Everolimus and Temsirolimus have both been approved by the FDA to treat advanced RCC.

## Conclusions

There is abundant evidence regarding the link between MS and RCC, and each component of MS may have a close causal association with RCC. Recent studies have generated exciting findings on the molecular basis of MS-associated RCC. Understanding the molecules involved may provide clues to prevent carcinogenensis in people with MS. Therapeutic interventions targeting these molecular mechanisms likewise manifest a positive perspective for the treatment of RCC. Further, because MS derives at least in part from an unhealthy lifestyle, maintaining a healthy lifestyle may be important in the prophylaxis of many cancers, including RCC.

## Competing interests

The authors declare that they have no competing interests.

## Authors’ contributions

GMZ and DWY conceived of the concept and searched references. GMZ and YZ drafted the manuscript. YZ provided the National Science Foundation of China (Grant No. NSFC 81001131). DWY revised the manuscript. All authors read and approved the final manuscript.
